# A prospective cohort study assessing the relationship between long-COVID symptom incidence in COVID-19 patients and COVID-19 vaccination

**DOI:** 10.1038/s41598-023-30583-2

**Published:** 2023-03-25

**Authors:** Bayan Abu Hamdh, Zaher Nazzal

**Affiliations:** 1 Department of Public Health, Faculty of Graduate Studies, An-Najah National University, Nablus, Palestine; 2 Department of Medicine, Faculty of Medicine and Health Sciences, An-Najah National University, Box 7707, Nablus, Palestine

**Keywords:** Infectious diseases, Diseases, Health care, Risk factors

## Abstract

Current studies about the long-term effects of COVID-19 show a wide range of symptoms. This prospective cohort study aimed to find the incidence of long-COVID symptoms and the associated risk factors. We followed 669 confirmed COVID-19 patients. Sociodemographic and clinical data were extracted from medical records and collected via semi-structured telephone interviews on days 10, 30, 60, and 90. The incidence of long-COVID symptoms was 41.6% (95% CI 37.8–45.4%). Females [aOR = 1.5 (95% CI 1.1–2.3)], the elderly [aOR = 4.9 (95% CI 2.0–11.3)], and those who required hospitalization [aOR = 5.0 (95% CI 1.3–3.7)] were at a higher risk of developing long-COVID. Patients with dyspnea at day 10 [aOR: 2.4 (95% CI 1.6–3.7] and fatigue at day 60 [aOR: 3.1 (95% CI 1.5–6.3] were also at risk. While non-vaccinated patients were almost seven times more likely to report long-COVID symptoms than vaccinated patients [aOR: 6.9 (95% CI 4.2–11.3)]. In conclusion, long-COVID was common among COVID-19 patients, with higher rates among females, older age groups, hospitalized patients, and those with dyspnea and fatigue, while vaccination provided protection. Interventions should educate health professionals, raise general public awareness about the risks and consequences of Long COVID, and the value of vaccination.

## Introduction

COVID-19 first appeared in China in December 2019 and rapidly spread worldwide, becoming a public health emergency and a global pandemic^1^. The disease was first reported in March 2020 in Palestine, and by October 2022 there were approximately 700,000 confirmed COVID-19 cases with 5700 deaths^2^.

In addition to ongoing COVID-19 outbreaks, healthcare providers are confronting the persistence of COVID-19 symptoms after the initial acute phase of infection^3^. Patients with COVID-19 who continue to complain of new, returning, or ongoing health problems 12 weeks post-infection that are not explained by an alternative diagnosis are described as post-COVID-19 conditions, long-COVID symptoms, or long-COVID syndrome^4^. The World Health Organization (WHO) definition includes the continuation or development of new symptoms 3 months after the initial SARS-CoV-2 infection (suspected or confirmed), with these symptoms lasting for at least 2 months with no other explanation^5^.

The frequency of long-COVID symptoms vary with some studies reporting that a quarter of COVID-19 patients experience symptoms 4–5 weeks after the positive test, while others report one in ten patients experiences symptoms after 12 weeks^4,6–9^. Several studies have described over 200 symptoms identified post-acute infection, with multiorgan involvement. The most common symptoms are fatigue, dyspnea, dysautonomia, postural orthostatic tachycardia syndrome, loss of taste and smell, insomnia, cough, headache, arthralgia, mental disorders, fever, hair loss, and gastrointestinal symptoms^3,10,11^. Long-COVID impacts both physical and mental well-being^12^, as well as functional and occupational status^13^, leading to prolonged disability with no return to the baseline health status for several months or years^14^. Furthermore, only a few of persons with Long COVID make a full recovery; 85% of Long COVID patients who had symptoms 2 months after infection still had symptoms 1 year later^8,15^.

Many theories have been proposed to explain its etiology, such as persistent reservoirs of SARS-CoV-2 in tissues, immunological dysregulation, autoimmunity, clotting and endothelial abnormalities, and dysfunctional signaling in the nervous system^8,16^. The risk factors that increase the occurrence of long COVID include older age, female gender, type 2 diabetes, certain autoantibodies, hospitalization, and certain socioeconomic conditions such as a lower income^17–19^.

The variety of COVID-19 vaccines appear to be effective in preventing infection or reducing the severity of symptoms^20,21^. However, their effect on the frequency of long COVID differs with some research reporting no effectiveness and others showing moderate protection^8,20–22^. A systematic review concluded that vaccination before SARS-CoV-2 infection decreased the risk of subsequent long-COVID infections^23^.

Studies on long COVID examine different populations in varied healthcare systems, where tests and examination equipment vary, as do the follow-up intervals. This study aims to measure the incidence of long-COVID symptoms among COVID-19 patients in Palestine seeking care in a government clinic and to assess the relationship between COVID-19 vaccination and the occurrence of long-COVID symptomatology.

## Methods

### Study design and population

We conducted a prospective cohort study that followed COVID-19 patients diagnosed between 16 September and 5 October 2021 in the Tulkarm Primary Health Care (PHC) Directorate of the West Bank with a positive COVID-19 test registered in the Health Information System. Phone interviews were conducted on days 10, 30, 60, and 90 to inquire about persisting symptoms. The study lasted until the end of January 2022 and included all adult patients over the age of 18 who had confirmed COVID-19 infection. Exclusion criteria included patients unable to answer the questionnaire or lost to follow-up. The Real Time-Polymerase Chain Reaction (RT-PCR) was used to confirm all COVID-19 cases in Palestine.

A total of 705 patients were identified, 36 were excluded: 33 left Palestine or failed to answer the phone, one had Down’s Syndrome and could not answer the questions, and two died. Six hundred and sixty-nine participants were included in the final analysis.

The Institutional Review Board of An-Najah National University [Reference #: Mast. Nov. 2021/33] approved the study. All procedures followed the Declaration of Helsinki guidelines. The Primary Healthcare Department of the Palestinian Ministry of Health granted permission to obtain essential information from the Health Information System. All patients were invited to participate, informed that their data would be used for research purposes, assured confidentiality, and gave verbal informed consent.

### Measurement tools

Demographic information including the date of infection, age, gender, and contact information were extracted from the patients’ electronic health records by the first author (BA). Follow-up clinical data were collected by phone using a semi-structured questionnaire derived from previous research on long-COVID symptoms^11,14,24^. Part one of the questionaire assessed patients’ smoking status, occupation, history of chronic illnesses, previous COVID-19 infection, and COVID-19 vaccination. The second part asked about the persistent or new emerging symptoms post-infection, like fever, cough, dyspnea, fatigue, loss of smell and taste, arthralgia, insomnia, brain fog, headache, and dyspnea. The first interview was conducted on day 10 to assess patients for persistent symptoms or new emerging symptoms. Follow up phone interviews were done on days 30, 60 and 90 using data from the prior interviews and asking about persistent or new symptoms.

Dyspnea was evaluated using the modified medical research council (mMRC) scale^25^. It is the most commonly validated scale for evaluating dyspnea in chronic respiratory diseases^26^, with five items describing nearly the entire spectrum of dyspnea, from none (Grade 0) to near-total incapacity (Grade 4)^25^. The mMRC was recorded as *No* for a score of zero and *Yes* for grades one through four. Fatigue was assessed with the Fatigue Assessment Scale (FAS), a valid questionnaire for assessing fatigue in patients with interstitial lung and other chronic lung diseases, and used to monitor fatigue in many diseases and conditions^27^. It has ten items; five examine physical fatigue and five mental fatigue on a scale that ranges from one to five. Higher scores indicate greater fatigue. FAS items 1, 2, 4, 5, and 10 assess physical fatigue, while items 3, and 6 through 9 assess mental fatigue. Item 4 and item 10 are scored in reverse. The total score is calculated by summing the scores for each question. It ranges between 10 and 50, with a score of < 22 indicating no fatigue and a score of  ≥ 22 as positive for fatigue^14,27,28^. The subscores for physical and mental fatigue have not been validated. The Arabic version of FAS is valid and reliable^29^.

### Statistical analysis

IBM SPSS Statistics software version 21 (IBM Corp., Armonk, NY, USA) was used for data analysis. Frequency distributions and proportions summarize the categorical variables with the chi-square test used to examine the relationships between categorical variables. The Kolmogorov–Smirnov test was emplyed to determine if the continuous variables were normally distributed and then data were summarized using means and standards deviation (SD). Finally, multivariate logistic regression was performed to identify factors independently associated with the long-COVID symptoms. The multivariate model included significant variables in the bivariate analysis, and the results were displayed as adjusted odds ratios (aOR) and 95% confidence intervals (95% CI). A P-value less than 0.05 was used to determine significance.

## Results

### Patients’ characteristic

Forty three percent of the cohort was male (288). The mean age was 35. 9 ± 11.5 years, with 371 (55.5%) employed and 191 (28.6%) smokers. Hypertension was the most common comorbidity, reported in 128 (18.2%) patients. Forty-five (6.7%) were previously infected with COVID-19, and 45 (6.7%) were hospitalized (Table 1).

**Table 1:** Background and clinical characteristics of the participants (n = 669).

Characteristics	Frequency	Percentage	Mean ± SD
Age			35.9 ± 11.5
18–29	239	35.7%	
30–39	185	27.7%	
40–49	134	20.0%	
≥ 50	111	16.6%	
Gender			
Male	288	43.0%	
Female	381	57.0%	
Marital status			
Married	500	74.7%	
Single	169	25.3%	
Educational level			
Diploma or higher	365	54.6%	
Secondary or primary school	304	45.4%	
Employment status			
Unemployed	298	44.5%	
Employed	371	55.5%	
Smoking			
Yes	191	28.6%	
No	478	71.4%	
Past medical history			
Hypertension	122	18.2%	
Diabetes mellitus	70	10.5%	
Ischemic heart disease	53	7.9%	
Respiratory diseases	49	7.3%	
Others*	58	8.7%	
Hospital admission	45	6.7%	

*Hypo/hyperthyroidism, inflammatory bowel disease, breast cancer, hepatitis.

### COVID-19 vaccination

Prior to infection, 274 (41.0%) participants were vaccinated against COVID-19 with 64 (23.4%) receiving only one dose. Patients received vaccines manufactured by Pfizer, Sputnik Light, and Moderna, 119 (17.8%), 85 (12.7%), and 25 (3.7%), respectively (Table 2).

**Table 2:** Vaccination status and type of COVID-19 vaccine of the study participants (n = 669).

	Frequency	Percentage
Vaccination status		
Yes	274	41.0%
No	395	59.0%
Type of vaccine		
Pfizer	119	17.8%
Sputnik light	85	12.7%
Moderna	25	3.7%
Sputnik-V	16	2.4%
Sinopharm	13	1.9%
AstraZeneca	16	2.4%
Number of doses (n = 274)		
One dose	64	23.4%
Two doses	210	76.6%
Previous COVID-19		
Yes	45	6.7%
No	624	93.3%

### Clinical characteristics

Table 3 presents the most common symptoms at days 10, 30, 60, and 90. Dyspnea (38.7%), cough (31.8%), insomnia (31.5%), and fatigue (29.3%) were the most common symptoms reported by patients on day 10. Dyspnea and fatigue were still the most common symptoms reported on day 90, 134 (20.0%) and 100 (14.9%), respectively.

**Table 3:** Frequency of COVID-19 signs and symptoms at day 10, day 30, day 6, and day 90 (n = 669).

Post COVID-19 symptoms	Day 10	Day 30	Day 60	Day 90
Dyspnea	259 (38.7%)	204 (30.5%)	170(25.4%)	134 (20.0%)
Fatigue	196 (29.3%)	295 (44.1%)	193 (28.8%)	100 (14.9%)
Physical fatigue				
Mental fatigue				
Headache	123 (18.4%)	79 (11.8%)	73 (10.9%)	60 (9.0%)
Insomnia	211 (31.5%)	85 (12.7%)	66 (9.9%)	42 (6.3%)
Ageusia	171 (25.6%)	95 (14.2%)	80 (12.0%)	49 (7.3%)
Anosmia	43 (6.4%)	35 (5.2%)	34 (5.1%)	31 (4.6%)
Brain fog	38 (5.7%)	33 (4.9%)	24 (3.6%)	16 (2.4%)
Cough	213 (31.8%)	41 (6.1%)	13 (1.9%)	12 (1.8%)
Dizziness	165 (10.5%)	36 (5.4%)	9 (1.3%)	13 (1.9%)
GI symptoms	106 (15.8%)	5 (0.7%)	1 (0.1%)	1 (0.1%)
Anorexia	104 (15.5%)	6 (0.9%)	2 (0.3%)	3 (0.4%)
Arthralgia	99 (14.8%)	18 (2.7%)	8 (1.2%)	4 (0.6%)
Fever	72 (10.8%)	2 (0.3%)	0 (0.0%)	0 (0.0%)

### Long-COVID symptoms

After 90 days, 278 patients [41.6%; (95% CI 37.8–45.4%)] were categorized as having long-COVID symptoms. In bivariate analysis, females, older age groups, having comorbidities, requiring hospitalization, non-vaccinated, dyspnea symptoms at day 10, and reports of fatigue at day 30 and day 60 were more likely to develop long-COVID (P < 0.001 for all). A multivariable analysis was conducted to identify factors independently associated with the outcome. Females were 1.6 times more likely to develop long-COVID symptoms than males [aP value: 0.019, aOR = 1.6 (95% CI 1.1–2.4)]. Older age groups were more likely, as well as patients over 50 who were nearly five times likelier than those aged 18–29 [aP value: < 0.001, aOR = 4.9 (95% CI 2.0–11.3)]. Patients hospitalized due to COVID-19 were at five times greater risk of suffering from long-COVID symptoms [aP value: < 0.001, aOR: 5.0 (95% CI 1.3–18.9)]. Furthermore, dyspnea on day 10 and fatigue on day 60 predicted long-COVID. Patients with dyspnea on day 10 were 2.4 times more likely to develop the long-COVID [aP value: < 0.001, aOR: 2.4 (95% CI 1.6–3.7)]. Those with fatigue on day 60 were nearly three times more likely to develop the long-COVID symptoms [aP value: < 0.001, aOR: 2.9 (95% CI 1.6–5.1]. On the other hand, vaccination was protective; non-vaccinated patients were almost seven times more likely to develop long-COVID symptoms than vaccinated patients [aP value: < 0.001, aOR: 6.9 (95% CI 4.2–11.3)] (Table 4).

**Table 4:** Bivariable and multivariable analysis of factors related to long-COVID symptoms (n = 669).

	Long-COVID symptoms frequency (%)		P value*	Multivariable analysis	
	Yes	No		aOR (95% CI)	aP value
Gender					
Male^†^	92 (31.9%)	196 (68.1%)	< 0.001	1.6 (1.1–2.4)	0.019
Female	186 (48.8%)	195 (51.2%)		1	
Age group					
18–29^†^	55 (23.0%)	184 (77.0%)	< 0.001	1	
30–39	76 (41.1%)	109 (58.9%)		2.2 (1.3–3.7)	0.003
40–49	82 (61.2%)	52 (38.8%)		4.9 (2.4–10.1)	< 0.001
≥ 50	65 (58.6%)	46 (41.4%)		4.9 (2.0–11.3)	< 0.001
Educational level					
Primary	20 (7.2%)	21(5.4%)	0.247		
Secondary	110 (39.6%)	153 (39.1%)			
Diploma	20 (7.2%)	17 (4.3%)			
Bachelors and more	128 (46.0%)	200 (51.2%)			
Employment status					
Unemployed	132 (44.3%)	166 (55.7%)	0.412		
Employed	146 (39.4%)	225 (61.6%)			
Smoking					
Yes	79 (41.4%)	112 (58.6%)	0.949		
No	199 (41.6%)	279 (58.4%)			
Comorbidities					
Yes	141 (58.7%)	99 (41.3%)	< 0.001	1.3 (0.73–2.1)	0.408
No^†^	137 (31.9%)	292 (68.1%)		1	
Hospital admission					
Yes	42 (93.3%)	3 (6.7%)	< 0.001	5.0 (1.3–18.9)	.019
No^†^	236 (37.8%)	388 (62.2%)		1	
Dyspnea on day 10					
Yes	111 (39.9%)	299 (76.5%)	< 0.001	2.4 (1.6–3.7)	< 0.001
No^†^	167 (60.1%)	92 (23.5%)		1	
Previous COVID-19					
Yes	19 (42.2%)	26 (57.8%)	0.925		
No	259 (41.5%)	365 (58.5%)			
COVID-19 vaccination					
Yes^†^	57 (20.8%)	217 (79.2%)	< 0.001	1	
No	221 (55.9%)	174 (44.1%)		6.9 (4.2–11.3)	< 0.001
Fatigue at day 30					
Yes	190 (68.3%)	105 (26.9%)	< 0.001	1.5 (0.86–2.6)	0.188
No^†^	88 (31.7%)	286 (73.1%)		1	
Fatigue at day 60					
Yes	150 (54.0%)	43 (11.0%)	< 0.001	3.1 (1.5–6.3)	0.002
No^†^	128 (46.0%)	348 (89.0%)		1	

*aP-value* adjusted P value, *aOR* adjusted odds ratio, *CI* confidence interval.

^†^Reference group, *Chi-square test.

## Discussion

This cohort study followed patients diagnosed with COVID-19 to assess long-term symptoms that persisted 90 days after the onset of the disease. Almost half of the affected sample was between 30 and 49 years old, and the majority were female. These findings are consistent with other studies indicating that COVID-19 is more prevalent in females and those ages 30 to 60 years^14,30,31^.

One-third of the patients complained of dyspnea, fatigue, loss of taste or smell, and cough on day 10. These symptoms are commonly reported as a clinical manifestation of acute COVID-19^32–34^. An online cross-sectional study in Saudi Arabia found that 73% of patients complained of fatigue and weakness 7 to 8.1 days after infection, and 66% had musculoskeletal pain, headache, loss of smell, cough, and loss of appetite^35^. Our findings of persistant dyspea and fatigue in addition to headaches, loss of smell and taste, sleep disturbance, brain fog and cough align with other studies. Tabacof et al. found that within a year of infection, the most common ongoing symptoms were fatigue (82%), brain fog (67%), and headache (60%)^24^. Another Nigerian study found that 2 weeks after being released from isolation, more than one-third of patients had persistent fatigue (12.8%), headache (12.8%), and chest pain (9.8%)^31^.

Although most COVID-19-infected patients recover within a few weeks after the acute infection, 40.6% of our sample continued to have at least one symptom on day 90. The rates of long-COVID symptomatology varies in the literature. For example, a Mediterranean cohort study found that approximately 50% of patients had persistent symptoms 10–14 weeks after the first episode^36^, while a Jordanian study showed that 71.8% of patients still have one physical or psychological symptom 3 months or more after the first infection^37^. In two German studies, 14.2% of patients developed post-COVID-19 symptoms^38^, whereas 27.8% of non-hospitalized COVID-19 patients reported at least one symptom 4 months after the initial symptom^3^. The differing results could be attributed to the various types of studies used, the length of follow-up, vaccination rates, and healthcare system differences. To date, no particular test is used to confirm Long-COVID symptoms, and the full impact of COVID-19 on patients is yet to be understood.

The literature is mixed on the risk factors for long-COVID. A prospective cohort study in revealed that females and patients with comorbidities were at higher risk of developing long-COVID symptoms^17^, consistent with our findings although comorbidities were not significant at the multivariate level. A Jordan study found that female patients, older patients, and patients with comorbidities had a significantly increased risk of developing long-COVID^37^. However, a Nigerian study did not find an association between age, sex, presence of hypertension, diabetes, or multiple comorbidities and experiencing persistent post-COVID-19 symptoms^31^. We found that older patients were five time more likely than younger patients to develop long-COVID.

Studies show hospitalization to be a significant risk factor for developing long-COVID, in agreement with our findings. Pérez-González et al. and Hedberg P et al. demonstrated that hospitalized patients were more likely to have persistent symptoms and post COVID conditions than outpatients^18,39^. Asadi-Pooya et al. found that the number of days spent in the hospital was significantly correlated with long-COVID symptoms 3 months after infection^40^.

As we found, fatigue is present with all ages, both sexes, and even in healthier patients^8^, and as we found one of the most common persistent symptoms with post-COVID-19. Carvalho-Schneider et al. demonatrated a significant association between dyspnea in the early presentation of the infection and persistent symptoms^34^ which is in agreement with our findings. Another study showed that 70% of COVID-19 hospitalized patients complained of fatigue and/or dyspnea 7 months post discharge^41^.

Unvaccinated patients were seven times more at risk of developing long-COVID than vaccinated patients in our study. However, vaccine effects on long-COVID vary in the literature. This may be related to different study methodology, time betweein immunization and infection, and long-COVID definitions^8^. Taquet et al. observed no significant difference in the development of long COVID between vaccinated and unvaccinated persons^22^. Other studies show that vaccines offer partial protection. For example, a large UK study with 1.2 million participants found that long-COVID symptoms were halved in vaccinated patients^21^. Another study revealed that patients vaccinated with two doses were 41% less likely to develop long-COVID symptoms^20^. These findings support the vaccine’s effectiveness in preventing the disease, limiting its spread, and mitigating its severity. The importance of promoting vaccination at the population level is also important in Palestine given that vaccination uptake among the general population by October 2022 was 36.6%^42^.

One of the strenghts of our study was the prospective follow-up based on the WHO definition of long-COVID. Because patients could be reached by phone and completed the entire interview questionaire on days 10, 30, 60, and 90, we documented and assessed new and emerging symptoms. Study limitations included self-reported data from telephone interviews rather than physical examinations, raising the concern of information bias and the ability to evaluate all comorbidities, functional impairments, and complications. Second, although a small number of patients were not followed for 90 days due to travel outside Palestine or inability to reach them by phone, this may have contributed to selection bias. Third, we could not examine all relevant risk factors and comorbidities, and encourage future researchers to do this. Lastly, while we based our 90-day follow-up time on the WHO definition of long-COVID, following patients for more than 90 days would be beneficial. We were unable to do so due to limited resources.

## Conclusion

Long-COVID symptoms incidence is high among COVID-19 patients, especially women, ages greater than 30, and those hospitalized for the infection. The most common persistent symptoms were dyspnea, fatigue, headache, insomnia, and loss of smell or taste. Patients with dyspnea on day 10, and fatigue on day 60 were at risk for developing long-COVID symptoms. COVID-19 vaccination appears to be protective against developing long-COVID symptoms.

To ensure that patients with long-COVID symptoms and associated illnesses receive high quality care, health professionals must be educated on the disease. In addition, education campaigns to inform the public about the risks and consequences of Long COVID and to promote vaccination is imperative. Centers of excellence should be established to provide high-quality, culturally competent healthcare, to conduct research on this evolving disease, and to educate primary care professionals who work on the frontlines.

## Data Availability

All data generated or analyzed during this study are included in this published article.

## References

[1] Lu H, Stratton CW, Tang Y-W (2020). Outbreak of pneumonia of unknown etiology in Wuhan, China: The mystery and the miracle. J. Med. Virol..

[2] occupied Palestinian territory, including east Jerusalem: WHO Coronavirus Disease (COVID-19) Dashboard With Vaccination Data | WHO Coronavirus (COVID-19) Dashboard With Vaccination Data. https://covid19.who.int/region/emro/country/ps (Accessed 17 October 2022) (2022).

[3] Augustin M, Schommers P, Stecher M (2021). Post-COVID syndrome in non-hospitalised patients with COVID-19: A longitudinal prospective cohort study. Lancet Reg. Heal.—Eur..

[4] CDC. Post-COVID Conditions: Information for Healthcare Providers. *U.S. Department of Health & Human Services* 2021; 2019–2021.

[5] World Health Organization. Post COVID-19 condition (Long COVID). https://www.who.int/europe/news-room/fact-sheets/item/post-covid-19-condition (Accessed 8 December 2022) (2022).

[6] Huang C, Huang L, Wang Y (2021). 6-month consequences of COVID-19 in patients discharged from hospital: A cohort study. Lancet.

[7] Rajan S, Khunti K, Alwan N, et al. In the wake of the pandemic: Preparing for Long COVID. *In the wake of the pandemic: Preparing for Long COVID*. https://apps.who.int/iris/bitstream/handle/10665/339629/Policy-brief-39-1997-8073-eng.pdf (Accessed 8 August 2022) (2021).

[8] Davis HE, McCorkell L, Vogel JM, et al. Long COVID: major findings, mechanisms and recommendations. *Nat Rev Microbiol*. Epub ahead of print 2023. DOI: 10.1038/s41579-022-00846-2. 10.1038/s41579-022-00846-2 PMC9839201 36639608

[9] Collaborators GB, of DLC.  (2022). Estimated global proportions of individuals with persistent fatigue, cognitive, and respiratory symptom clusters following symptomatic COVID-19 in 2020 and 2021. JAMA.

[10] Tenforde MW, Kim SS, Lindsell CJ (2020). Symptom duration and risk factors for delayed return to usual health among outpatients with COVID-19 in a multistate health care systems network—United States, March–June 2020. MMWR Morb. Mortal Wkly. Rep..

[11] Halpin SJ, McIvor C, Whyatt G (2021). Postdischarge symptoms and rehabilitation needs in survivors of COVID-19 infection: A cross-sectional evaluation. J. Med. Virol..

[12] Righi E, Mirandola M, Mazzaferri F (2022). Determinants of persistence of symptoms and impact on physical and mental wellbeing in Long COVID: A prospective cohort study. J. Infect..

[13] Dryden M, Mudara C, Vika C (2022). Post-COVID-19 condition 3 months after hospitalisation with SARS-CoV-2 in South Africa: A prospective cohort study. Lancet Glob. Health.

[14] Davis HE, Assaf GS, McCorkell L, et al. Characterizing Long COVID in an International Cohort: 7 Months of Symptoms and Their Impact. *SSRN Electron J*. Epub ahead of print 2021. DOI: 10.2139/ssrn.3820561. 10.1016/j.eclinm.2021.101019 PMC8280690 34308300

[15] Tran V-T, Porcher R, Pane I (2022). Course of post COVID-19 disease symptoms over time in the ComPaRe long COVID prospective e-cohort. Nat. Commun..

[16] Bisaccia G, Ricci F, Recce V, et al. Post-Acute Sequelae of COVID-19 and Cardiovascular Autonomic Dysfunction: What Do We Know? *J Cardiovasc Dev Dis*; 8. Epub ahead of print November 2021. DOI: 10.3390/jcdd8110156. 10.3390/jcdd8110156 PMC8621226 34821709

[17] Pazukhina E, Andreeva M, Spiridonova E (2022). Prevalence and risk factors of post-COVID-19 condition in adults and children at 6 and 12 months after hospital discharge: A prospective, cohort study in Moscow (Stop COVID). SSRN Electron. J..

[18] Pérez-González A, Araújo-Ameijeiras A, Fernández-Villar A (2022). Long COVID in hospitalized and non-hospitalized patients in a large cohort in Northwest Spain, a prospective cohort study. Sci. Rep..

[19] Su Y, Yuan D, Chen DG (2022). Multiple early factors anticipate post-acute COVID-19 sequelae. Cell.

[20] Ayoubkhani D (2022). Risk of long COVID in people infected with severe acute respiratory syndrome coronavirus 2 after 2 doses of a coronavirus disease 2019 vaccine: community-based, matched cohort study. Open Forum Infect. Dis..

[21] Antonelli M, Penfold RS, Merino J (2022). Risk factors and disease profile of post-vaccination SARS-CoV-2 infection in UK users of the COVID Symptom Study app: A prospective, community-based, nested, case-control study. Lancet Infect. Dis..

[22] Taquet M, Dercon Q, Harrison PJ (2022). Six-month sequelae of post-vaccination SARS-CoV-2 infection: A retrospective cohort study of 10,024 breakthrough infections. Brain Behav. Immun..

[23] Notarte KI, Catahay JA, Velasco JV, et al. Impact of COVID-19 vaccination on the risk of developing long-COVID and on existing long-COVID symptoms: A systematic review. *eClinicalMedicine*; 53. Epub ahead of print 1 November 2022. DOI: 10.1016/j.eclinm.2022.101624. 10.1016/j.eclinm.2022.101624 PMC9417563 36051247

[24] Tabacof L, Tosto-Mancuso J, Wood J (2022). Post-acute COVID-19 syndrome negatively impacts physical function, cognitive function, health-related quality of life, and participation. Am. J. Phys. Med. Rehabil..

[25] Hajiro T, Nishimura K, Tsukino M (1998). Analysis of clinical methods used to evaluate dyspnea in patients with chronic obstructive pulmonary disease. Am. J. Respir. Crit. Care Med..

[26] Casanova C, Marin JM, Martinez-Gonzalez C (2015). Differential effect of modified medical research council dyspnea, COPD assessment test, and clinical COPD questionnaire for symptoms evaluation within the new GOLD staging and mortality in COPD. Chest.

[27] Hendriks C, Drent M, Elfferich M (2018). The fatigue assessment scale: Quality and availability in sarcoidosis and other diseases. Curr. Opin. Pulm. Med..

[28] Hussain N, Samuelsson CM, Drummond A (2022). Prevalence of fatigue at one-year follow-up from the Gothenburg recovery and rehabilitation after COVID-19 and intensive care unit study. Sci. Rep..

[29] Al-Hanbali S, Aljasser A, Aboudi O (2022). Establishing the reliability and the validity of the Arabic translated versions of the effort assessment scale and the fatigue assessment scale. Int. J. Audiol..

[30] da Prestes GS, Simon CS, Walz R (2022). Respiratory outcomes after 6 months of hospital discharge in patients affected by COVID-19: A prospective cohort. Front. Med..

[31] Osikomaiya B, Erinoso O, Wright KO (2021). ‘Long COVID’: Persistent COVID-19 symptoms in survivors managed in Lagos State Nigeria. BMC Infect. Dis..

[32] Ferreira-Santos D, Maranhão P, Monteiro-Soares M (2020). Identifying common baseline clinical features of COVID-19: A scoping review. BMJ Open.

[33] Olumade TJ, Uzairue LI (2021). Clinical characteristics of 4499 COVID-19 patients in Africa: A meta-analysis. J. Med. Virol..

[34] Carvalho-Schneider C, Laurent E, Lemaignen A (2021). Follow-up of adults with noncritical COVID-19 two months after symptom onset. Clin. Microbiol. Infect..

[35] Khodeir MM, Shabana HA, Rasheed Z (2021). COVID-19: Post-recovery long-term symptoms among patients in Saudi Arabia. PLoS ONE.

[36] Moreno-Pérez O, Merino E, Leon-Ramirez JM (2021). Post-acute COVID-19 syndrome incidence and risk factors: A mediterranean cohort study. J. Infect..

[37] Almasri M-S, Alqaisi R, Al-Shagahin M (2022). Risk factors and characterization of post-COVID-19 syndrome in Jordan. Iproceedings.

[38] Donnachie E, Hapfelmeier A, Linde K (2022). Incidence of post-COVID syndrome and associated symptoms in outpatient care in Bavaria, Germany: A retrospective cohort study using routinely collected claims data. BMJ Open.

[39] Hedberg P, Granath F, Bruchfeld J (2023). Post COVID-19 condition diagnosis: A population-based cohort study of occurrence, associated factors, and healthcare use by severity of acute infection. J. Intern. Med..

[40] Asadi-Pooya AA, Akbari A, Emami A (2021). Risk factors associated with long covid syndrome: A retrospective study. Iran. J. Med. Sci..

[41] Fernández-De-las-Peñas C, Palacios-Ceña D, Gómez-Mayordomo V (2022). Fatigue and dyspnoea as main persistent post-COVID-19 symptoms in previously hospitalized patients: Related functional limitations and disability. Respiration.

[42] covidvax.live – Palestine. https://covidvax.live/location/pse (Accessed 13 October 2022).

